# Unique amplification of BCR-ABL1 gene fusion in a case of T-cell acute lymphoblastic leukemia

**DOI:** 10.1186/s13039-017-0340-6

**Published:** 2017-10-26

**Authors:** Rima Koka, Najeebah A. Bade, Edward A. Sausville, Yi Ning, Ying Zou

**Affiliations:** 1 Department of Pathology, University of Maryland School of Medicine Baltimore, 22 S. Greene St NBW53, Baltimore, MD 21201 USA; 2 Department of Medicine, University of Maryland School of Medicine, 655 W Baltimore S, Baltimore, MD 21201 USA; 3 University of Maryland Marlene and Stewart Greenebaum Cancer Center, Baltimore, MD USA; 4 Department of Pathology, Johns Hopkins Hospital, Baltimore, MD USA

**Keywords:** T-lymphoblastic leukemia, BCR/ABL, Gene amplification, Isodicentric chromosome 22, Derivative chromosome 9

## Abstract

**Background:**

ABL1 gene translocations can be seen in precursor T-acute lymphoblastic leukemia (T-ALL). The typical translocation partner is the NUP214 gene. BCR-ABL translocations are relatively rare in this entity. Furthermore, while there have been unique patterns of amplification noted among the NUP214-ABL fusion genes, there have been few such reports among cases with BCR-ABL fusion genes.

**Case presentation:**

Here we report a unique case of a 44-year old patient with T-ALL in which the blasts demonstrated a derivative chromosome 9 involving a 9;22 translocation and a dicentric Philadelphia chromosome 22 with a homogeneously staining region at the interface of the 9;22 translocation, leading to BCR-ABL1 gene amplification. Fluorescence in-situ hybridization (FISH) showed abnormal BCR/ABL1 fusions with the BCR-ABL1 gene amplification in 48% of the interphase cells analyzed. The translocation was confirmed by SNP array.

**Conclusions:**

We present a novel derivative chromosome 9 that shows BCR-ABL gene fusion along with a dicentric Philadelphia chromosome 22 with BCR-ABL1 gene amplification. This is a unique pattern of BCR-ABL fusion which has never been described in T-ALL. It is significant that the patient responded to standard treatment with the CALGB 10403 protocol and supplementation with a tyrosine kinase inhibitor. Identification of additional patients with this pattern of BCR-ABL fusion will allow for enhanced risk assessment and prognostication.

## Background

Precursor T-lymphoblastic lymphoma/leukemia is a malignancy derived from T-cell precursors. It can manifest as a mass-forming lesion, designated as lymphoma, of the thymus and/or lymph nodes or as leukemia, with involvement of the peripheral blood and bone marrow. The distinction between lymphoma and leukemia is somewhat arbitrary, particularly since all cases have some involvement of the bone marrow; however, the presence of greater than 25% lymphoblasts in the bone marrow should be classified as leukemia [1]. The lymphoblasts are characterized by small to medium-sized cells with moderately condensed to dispersed chromatin, indistinct nucleoli and scant basophilic cytoplasm. Cytoplasmic vacuoles may also be seen. There is no morphologic distinction between T-lymphoblasts from the B-lymphoblasts seen in precursor B-lymphoblastic lymphoma/leukemia (B-ALL). In order to distinguish T-lymphoblasts from B-lymphoblasts, ancillary testing using flow cytometry and/or immunohistochemistry is necessary. The most lineage specific marker is cytoplasmic CD3 although other T-cell markers CD2, CD4, CD8, CD5, CD7 and CD8 can be expressed, often in a pattern that reflects the stage of intrathymic differentiation. In order to distinguish from a mature T cell malignancy, expression of one or more immature markers CD1a, CD34 or TdT is needed. Approximately 19–32% of cases aberrantly express myeloid markers CD13 and CD33 while CD117 may be expressed in cases with activating FLT-3 mutations [2–4]. Precursor T-lymphoblastic lymphoma (T-LBL) constitutes 85–90% of all lymphoblastic lymphomas while precursor T-lymphoblastic leukemia (T-ALL) accounts for approximately 15% and 25% of childhood and adult lymphoblastic leukemias respectively [1]. Both T-LBL and T-ALL demonstrate slight male predominance.

An abnormal karyotype is found in approximately 50% of T-ALL/LBL cases with a significant proportion involving T-cell receptor (TCR) genes; the alpha and delta loci at 14q11.2, the beta locus at 7q35 and the gamma locus at 7p14–15 partner with a variety of genes [5–8]. These translocations often lead to transcriptional dysregulation of the partner gene. Translocations not involving the TCR genes are rare in T-ALL/LBL. One of these unusual translocations involves the balanced translocation of the Abelson (ABL1) oncogene at chromosome 9q34 to BCR on chromosome 22q11 to produce what is known as the Philadelphia chromosome. In this report, we present a unique case of T-ALL/LBL with amplification of BCR-ABL1 fusion gene in an isodicentric derivative Philadelphia chromosome 22 (idic der (22) t (9;22) (q34;q11.2) hsr (9;22)) with a homogeneously staining region at the interface of the 9;22 translocation.

## Case presentation

A 44 year old Caucasian female with a history of hypertension, obstructive sleep apnea, and morbid obesity, presented to the hospital with severe bony pains, worsening fatigue, and shortness of breath. She had a 25 pound weight loss over the past 1 month as well as drenching night sweats. She did not smoke cigarettes, drink alcohol or use illicit drugs. She had not started any new medications recently.

She was found to have a white blood cell count of 109,500/mcL (differential: 31% segmented neutrophils, 25% myelocytes, 21% lymphocytes, 2% monocytes, 7% eosinophils, 14% blasts), hemoglobin 8.5 g/dL, and platelet count of 111,000/mcL. Her creatinine was 1.6 mg/dL, serum calcium 14.8 mg/dL, LDH 581 units/L, and uric acid was 18.6 mg/dL. Peripheral blood smear showed marked leukocytosis with several immature myeloid precursors and blasts. The blasts were intermediate in size with loosely clumped chromatin, inconspicuous nucleoli and scant basophilic cytoplasm. The bone marrow biopsy and clot section demonstrated a hypercellular (90%) marrow for the age of the patient. Maturing granulopoiesis and erythropoiesis were scant secondary to replacement by a monotonous population of immature cells. Flow cytometric analysis confirmed the presence of an abnormal T-cell population expressing cytoplasmic CD3, CD2, CD4, CD5, CD7, CD8, CD45, and CD1a. Surface CD3 and TdT expression levels were dim to negative.

**Fig. 1:**
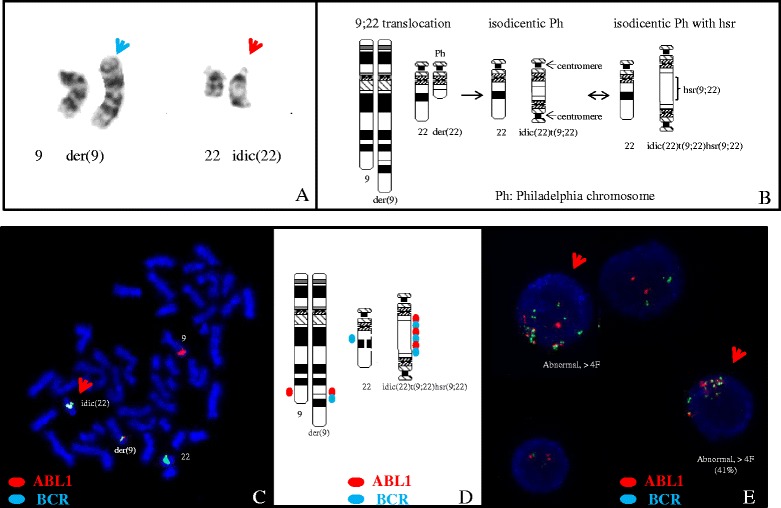
Cytogenetic analysis on bone marrow sample of the patient at diagnosis. **a** Partial karyogram showing chromosomes 9 and 22 including a normal chromosome 9, a derivative chromosome 9 (green arrow), a normal chromosome 22, and an isodicentric Philadelphia chromosome 22 (red arrow). **b** Ideograms of G-banding patterns for chromosomes 9 and 22 showing the formation of the isodicentric Philadelphia chromosome 22 with hsr. **c** Metaphase-FISH using dual-color dual-fusion *BCR-ABL1* probes showing multiple copies of *BCR-ABL1* fusions in the isodicentric Philadelphia chromosome 22 (red arrow). **d** Ideograms of FISH signals for *BCR* and *ABL1* genes showing their distributions. **e** Interphase-FISH using dual-color dual-fusion *BCR-ABL1* probes showing multiple copies of *BCR-ABL1* fusion signals in 41% of interphase nucleus analyzed (red arrows)

Twenty metaphase cells from three cultures were analyzed by GTG banding at the 450 band level. 11 cells had a normal karyotype. Nine cells (45%) were abnormal with a derivative chromosome 9 involving a 9;22 translocation and a dicentric derivative chromosome 22 involving a 9;22 translocation and a homogeneously staining region at the interface of the 9;22 translocation, which was associated BCR-ABL1 gene amplification (Fig. 1a-b).

**Fig. 2:**
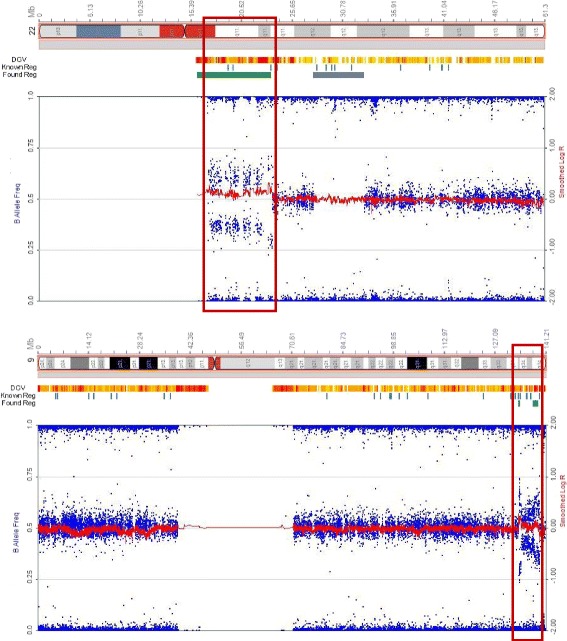
SNP microarray analysis of bone marrow sample of patient at diagnosis. SNP array analysis of chromosomes 9 (left) and 22 (right). Gain of 9q34 from genomic position (hg19) 133,624,374–139,394,573 (5.8 Mb) including ABL1, and gain of 22q11 from 16,114,244–23,648,478 (7.5 Mb) including BCR are indicated by the right shift of log R as well as change of B allele frequencies. Loss of heterozygosity at 22q12 (5 Mb) in 100% of the cells is observed, which most likely represents a constitutional change also seen in a few regions on other chromosomes in this patient

Fluorescence in-situ hybridization (FISH) was performed using a BCR/ABL1 dual color dual fusion probe set to rule out a 9;22 translocation. 200 interphase cells were examined. Abnormal BCR/ABL1 fusions with the BCR-ABL1 gene amplification were found in 48% of the interphase cells analyzed (Fig. 1c-e). Single nucleotide polymorphism (SNP) microarray analysis was performed and demonstrated gain of 9q34 from genomic position (hg19) 133,624,374–139,394,573 (5.8 Mb) including the ABL1 gene, and gain of 22q11 from 16,114,244–23,648,478 (7.5 Mb) including the BCR gene (Fig. 2). Additional findings included loss of heterozygosity at 22q12 (5 Mb), 4q, 5q, 6q, and 22q; all of these were noted in 100% of cells and thus, likely represent constitutional changes. Small changes of uncertain significance were also noted in 7q and 16p, but in less than 1 Mb.

Following diagnosis, hydroxyurea was used for cytoreduction to which the white blood cell count quickly responded. The patient was initially treated for tumor lysis syndrome with hydration and rasburicase, but eventually required continuous venovenous hemofiltration (CVVH) for acute renal failure. She also developed respiratory failure and required intubation.

Treatment was initiated with CALGB 10403 protocol which included Prednisone 60 mg/m2/day for days 1–28, vincristine 1.5 mg/m2 on days 1, 8, 15, and 22, daunorubicin 25 mg/m2 on days 1, 8, 15, and 22, and peg-asparaginase of 2500 units/m2 on day 4. She was also started on Imatinib at a dose of 400 mg by mouth daily on day 3 following initiation of chemotherapy. This dose was continued throughout her treatment. She also received intrathecal chemotherapy with cytarabine and methotrexate. Her course was complicated by septic shock which eventually led to initiation of pressor support. Throughout the hospitalization, the patient remained on CVVH. She also had chronic respiratory failure and required tracheostomy and continued ventilator support. A repeat bone marrow biopsy after count recovery showed no morphologic or immunophenotypic evidence of acute leukemia, was 40% cellular and had trilineage hematopoiesis; however, there was persistent neutropenia based on peripheral counts. Karyotype was 46XX and BCR-ABL PCR was negative.

Unfortunately, she eventually developed sacral decubitus ulcers that resulted in multiple infectious complications including persistent bacteremia and fungemia. Ultimately, the decision was made for comfort care. The patient passed away 86 days after admission although her leukemia was in complete remission.

## Discussion and conclusions

While translocation (9;22) (q34; q11) is most commonly associated with chronic myelogenous leukemia (CML), its presence has been noted in approximately 25% of de novo B-ALL cases, and confers a bad prognosis in the latter, although the use of Imatinib has mitigated this to a degree. In one study, the overall survival of the patients in the Imatinib cohort was 38% whereas the preimatinib group was 22% [9]. The fusion transcripts may yield either a p190 protein (minor breakpoint cluster; e1a2 bcr/abl junction) or a p210 protein (major breakpoint cluster; b2a2 or b3a2 bcr/abl junction). The former is most commonly associated with ALL while the latter is associated with CML. However, p210 transcripts can be seen in low levels in a p190 expressing ALL and vice versa [10, 11]. While this translocation has also previously been reported in T-ALL/LBL, it is exceedingly rare. Among all ALL/LBL cases that demonstrate translocation (9;22), only 2% have a T-cell phenotype [12–14]. Although rearrangement of ABL1 gene is seen commonly in T-ALL/LBL, its fusion partner is most often NUP214 rather than BCR. Interestingly, recent studies have identified that in the majority of NUP214-ABL1 positive cases, there is extrachromosomal amplification of the fusion protein that cannot be detected by conventional cytogenetics. This is due to the presence of extra copies of ABL1 within cytogenetically invisible units known as episomes [6, 12, 14].

Translocation (9;22) occurs very rarely in T-ALL/LBL with an estimated incidence of 2.3% in childhood T-ALL cases and 4.3% in adult T-ALL cases [1]. Although the literature regarding Philadelphia chromosome positive T-ALL is sparse and composed primarily of case reports, like its B-cell counterpart, Philadelphia chromosome positive T-ALL appears to carry a similarly bad prognosis [14–16]. Additionally, previous reports have demonstrated amplifications involving the NUP214-ABL1 fusion gene. The mechanisms of amplification have been heterogeneous. In some cases, there was episomal amplification alone while in others a few of the fusion genes were re-integrated into the chromosome with no subsequent amplification. In the cases with purely episomal fusion genes, there was no evidence of the fusion genes on conventional cytogenetic analysis. Therefore, in the absence of specific FISH analysis, these chromosomal fusions may never have come to light. This is of particular interest since there is some suggestion that tyrosine kinase inhibitors may have a role in the treatment of these patients [6]. While clinical experience is limited due to the rarity of these cases, the response of NUP214-ABL1-positive T-ALL patients to tyrosine kinase therapy seems to be highly variable [17–19]. This may be secondary to differences in the catalytic properties of NUP214-ABL1 and BCR-ABL1 as outlined by De Keersmaecker et al. The former was shown to have lower in vitro activity but with increased sensitivity to Imatinib in vitro [20]. Yet another mechanism of amplification was hypothesized to occur whereby the fusion genes within episomes are integrated into chromosomes but then undergo gene amplification [21]. The proposed hypothesis for how this amplification occurs is that the integration occurs downstream of a strong promoter [21].

In the present study, there is intrachromosomal evidence of the BCR-ABL1 fusion gene involving an isodicentric chromosome 22 in approximately 45% of cells. Therefore, we speculate that one of the models previously proposed for the NUP214-ABL1 fusion gene may apply in this case. The demonstration of this translocation in addition to amplification of the fusion gene has, to our knowledge, never been previously reported in T-ALL/LBL. Further detailed analyses of high-resolution sequencing data will provide insights into the mechanism of the BCR-ABL gene amplification in this novel derivative chromosome.

Additionally, due to the presence of this fusion gene, our patient was treated with traditional chemotherapy which was supplemented with a tyrosine kinase inhibitor. She appeared to respond to this therapy. While not relevant to this patient, future annotation of the course of such patients treated with abl-directed therapeutic agents would be of interest to determine if the risk of recurrence in patients with gene amplification is different from those with the fusion gene alone.

## Abbreviations

B-ALL: Precursor B-acute lymphoblastic leukemia
CVVH: Continuous venovenous hemofiltration
FISH: Fluorescence in-situ hybridization
SNP: Single nucleotide polymorphism
T-ALL: Precursor T-acute lymphoblastic leukemia
T-LBL: Precursor T-acute lymphoblastic lymphoma
